# Baseline-dependent clock offsets in VLBI data analysis

**DOI:** 10.1007/s00190-021-01579-5

**Published:** 2021-11-01

**Authors:** Hana Krásná, Frédéric Jaron, Jakob Gruber, Johannes Böhm, Axel Nothnagel

**Affiliations:** 1 Department of Geodesy and Geoinformation, Technische Universität Wien (TU Wien), Wiedner Hauptstraße 8-10, 1040 Vienna, Austria; 2 Astronomical Institute, Czech Academy of Sciences, Boční II 1401, 141 00 Prague, Czech Republic

**Keywords:** VLBI, IVS, CONT17, Baseline-dependent clock offsets

## Abstract

The primary goal of the geodetic Very Long Baseline Interferometry (VLBI) technique is to provide highly accurate terrestrial and celestial reference frames as well as Earth orientation parameters. In compliance with the concept of VLBI, additional parameters reflecting relative offsets and variations of the atomic clocks of the radio telescopes have to be estimated. In addition, reality shows that in many cases significant offsets appear in the observed group delays for individual baselines which have to be compensated for by estimating so-called baseline-dependent clock offsets (BCOs). For the first time, we systematically investigate the impact of BCOs to stress their importance for all kinds of VLBI data analyses. For our investigations, we concentrate on analyzing data from both legacy networks of the CONT17 campaign. Various aspects of BCOs including their impact on the estimates of geodetically important parameters, such as station coordinates and Earth orientation parameters, are investigated. In addition, some of the theory behind the BCO determination, e.g., the impact of changing the reference clock in the observing network on the BCO estimate is introduced together with the relationship between BCOs and triangle delay closures. In conclusion, missing channels, and here in particular at S band, affecting the ionospheric delay calibration, are identified to be the dominant cause for the occurrence of significant BCOs in VLBI data analysis.

## Introduction

Very Long Baseline Interferometry (VLBI) is a space geodetic technique which can be used to estimate the terrestrial and celestial reference frames together with all five Earth orientation parameters (EOP) (Schuh and Böhm 2012). It has a unique role in the maintenance of the celestial reference frame at radio frequencies with its current third realization ICRF3 (Charlot et al. 2020) and contributes significantly to a highly accurate and stable terrestrial reference frame (currently ITRF2014, Altamimi et al. 2016), in particular to its scale determination. VLBI is the only technique that provides the full set of Earth orientation parameters. It contributes uniquely to the EOP determination with direct measurements of nutation parameters and of the Earth rotation angle. The products of the International VLBI Service for Geodesy and Astrometry (IVS, Nothnagel et al. 2017) are essential for positioning and navigation on Earth and in space, and provide valuable information about interactions within the Earth system.

In addition to the parameters of interest for the geodetic analysis, auxiliary parameters have to be estimated as well. Relative clock offsets and their variations need to be determined to form the link between the time series of observations. In particular, we estimate a set of parameters describing the behaviour of the hydrogen maser clock at each station with an offset, linear trend and a quadratic term with respect to a station which is chosen as reference in the observing network. In addition to the clock parameters, other auxiliary parameters help to account for imperfect theoretical models such as effects of tropospheric refraction. Therefore, zenith wet delays and gradients are estimated at each station to describe the neutral part of the atmosphere around the station (Nilsson et al. 2013).

In this publication we deal with modelling of the atomic clocks and clock-like effects, and, in particular, with the so-called baseline-dependent clock offsets (BCOs). We investigate various aspects of BCOs including their impact on the estimates of geodetically important parameters. Furthermore, we formulate relationships between the individual clock parameters and we choose a systematic approach for the empirical tests. The reason for this publication is that the omission of BCOs in the functional VLBI model leads to significant deterioration of the geodetic VLBI results. For this reason, we describe the procedure for accounting of the BCOs in the analysis of VLBI data, and we show the impact of unmodelled BCOs on the geodetic parameters, such as station coordinates and Earth orientation parameters.

The article is structured on the basis that we accept the need for estimating BCOs without going into detail of possible causes since careful investigations of the reasons for BCOs will be subject of our future work. These forthcoming studies will be very useful in terms of feedback for the fringe fitting and pre-processing procedures of VLBI. After some initial considerations (Sect. 2), we describe the BCO estimation process and the caveats to consider (Sect. 3). In Sect. 4 we systematically analyse and discuss the implications of BCO in the CONT17 observing series (Behrend et al. 2020). In Sect. 5 we deal with the relationship between BCOs and triangle delay closures. We give our conclusions in Sect. 6.

## Initial considerations

The VLBI group delay observable $\uptau$ is only of value if a reference epoch is assigned to it. For reasons of computational practicality, most correlators produce group delays referred to the epoch when the wavefront passes the geocenter, called geocenter delays. In most group delay analysis packages for the estimation of geodetic parameters, the group delays need to be referred to the epoch, when the wavefront passes the first telescope of a baseline, providing the so-called baseline delay. The conversion between the two delays is a simple linear function using delay rate information (Corey 2000). The distinction between these two delays is elementary to considerations of forming triangle closures. If we discuss delay observables and triangle closures below, we always mean the total group delay where the conversion of the reference epoch has been dealt with implicitly.

Furthermore, it should be explained here that all delay and delay rate observables used in our investigations are the raw data from the correlator as stored in the respective databases. Ionosphere corrections, as provided by the nuSolve^1^ preprocessing (Bolotin et al. 2014), are applied as are the delay ambiguities. The latter are at the level of 50, 100 or 200 ns, which are several magnitudes larger than the closure delays encountered here, and blunders would be detectable easily. Only the ionosphere corrections, derived in a long-standing automatic process, produce additional noise which is discussed in Sect. 5.

In the following mathematical formulations the quantities such as wavefront passage epoch *T* or observed group delay $\uptau$ do change over time, baseline and radio source but the critical numbers such as the BCOs and closure delays vary only randomly. For this reason, the equations quoted below, strictly speaking, are only valid for one epoch. However, because the causes of the BCOs/mis-closures do not change beyond the source structure effects, they can be considered to be representative for the questions at hand.

Turning to the estimation of clock parameters, this is conceptually divided into two levels of modelling. The first is a simple second-order polynomial:

1 $$\begin{aligned} \varDelta \uptau (t)_\mathrm{clock}= & {} - (\mathcal {T}^A_0 + \mathcal {T}^A_1 \cdot (t - t_0) + \mathcal {T}^A_2 \cdot (t - t_0)^2 ) \nonumber \\&+ (\mathcal {T}^B_0 + \mathcal {T}^B_1 \cdot (t - t_0) + \mathcal {T}^B_2 \cdot (t - t_0)^2 ) \end{aligned}$$

with $\mathcal {T}_i$ denoting the coefficients of the polynomial and the indices *A* and *B* referring to the two telescopes. If the clock of one of these telescopes is chosen as the reference, the respective coefficients are set to equal zero. The second level of modelling are piece-wise linear offsets (PWLO) $\mathcal {T}^*_P$ (see Eq. 2) which serve to introduce a higher time resolution and which are superimposed onto the first level in the form of

2 $$\begin{aligned} \varDelta \uptau ^*_P(t) = \frac{ \mathcal {T}^*_P(t_i) - \mathcal {T}^*_P(t_{i-1})}{t_i - t_{i-1}}(t - t_{i-1}) \end{aligned}$$

by adding these parameters to each line of Eq. (1) independently (except for the reference clock). The $*$ denotes a wildcard for the station. The $t_i$ are the epochs of the adjacent offsets and *t* refers to the epoch of the observation. In standard VLBI analyses, the length of these segments is set to, e.g., 30 min or 1 h . While it would be sufficient to just use piece-wise linear offsets, the two levels are maintained because through the polynomials the PWLO should be made as flat as possible for the following reason: relative constraints of $\mathcal {T}^*_P(t_i) = \mathcal {T}^*_P(t_{i-1})$ with a certain level of variance can then be applied for stabilizing the polygon in case the number of observations in a time segment is not sufficient for a regular estimation.

Some VLBI analysis centers run a so-called *First Solution* before the main solution. In the first solution, the only estimates are the coefficients of the second-order polynomial (see Eq. (1)) plus constant zenith delays per station per session. The large clock values from the second-order polynomials of the first solution are subtracted from the observations before entering the main solution in order to avoid numerical problems mixing large clock estimates with the other centimeter-level estimates in the least-squares adjustment. Furthermore, first solutions are a useful tool to identify clock breaks or baseline dependent biases in the post-fit residuals which could be mitigated in the main solution.

It should be mentioned that the clock parameters can also be estimated as stochastic parameters in Kalman filter solutions (Herring et al. 1990; Soja 2016), but this option is not considered here. In completing these explanations, it should be stated that if there was perfect technical equipment at the stations and all of the stations observed exactly the same frequency bands of a source with negligible structure effects, the above-mentioned parameters would be sufficient to fit the theoretical VLBI model.

**Fig. 1:**
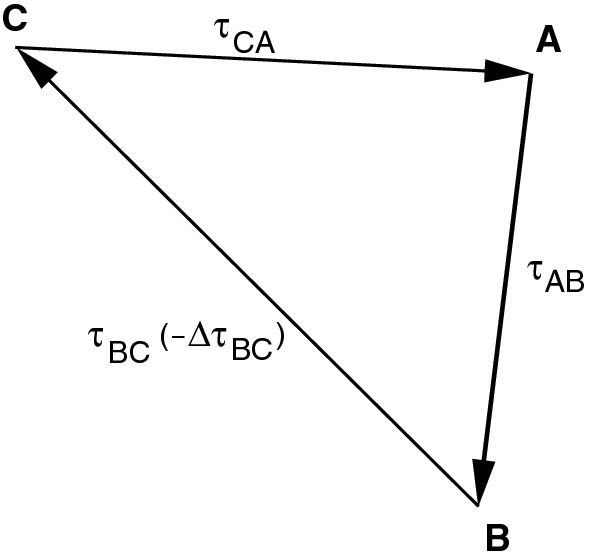
Schematic figure showing the relative geographical location of three stations *A*, *B*, and *C*, with baseline orientation indicated by the arrows, and the baseline delays $\uptau _{AB}$, $\uptau _{BC}$, and $\uptau _{CA}$ written beside each baseline together with a baseline clock offset $\varDelta \uptau _{BC}$

However, already in 1994 it was discovered that in addition to the parameters described above, in many cases significant offsets appear in the observed group delays for individual baselines as a whole which are compensated for by estimating BCOs (Ma et al. 1995). If *A* is the clock reference station in a triangle of stations *A*, *B*, *C*, and the observed total delays are $\uptau _{AB}$, $\uptau _{BC}$, and $\uptau _{CA}$, a baseline clock offset $\varDelta \uptau _{BC}$ manifests itself as a constant component of the observed delay $\uptau _{BC}$ (Fig. 1). The minus sign refers to the fact that for the delay $\uptau _{BC}'$ closing the triangle, the BCO $\varDelta \uptau _{BC}$ has to be subtracted. It should be emphasized that the delays $\uptau$ are not for individual observations but that all considerations of BCOs refer to all delay observables of a baseline of an observing session as a whole. Only the BCOs manifest themselves mainly as one constant each for the whole session. Strictly speaking, in principle, multiple BCOs could be estimated per baseline per session if needed in some special cases. In addition, the figure is valid for any BCO and independent of the origin of the BCOs. With our analysis concept with a *First Solution* to determine and apply a priori clock offsets, we do not corrupt the results of the BCO estimation because these values are applied station-wise and thus always compensate.

**Fig. 2:**
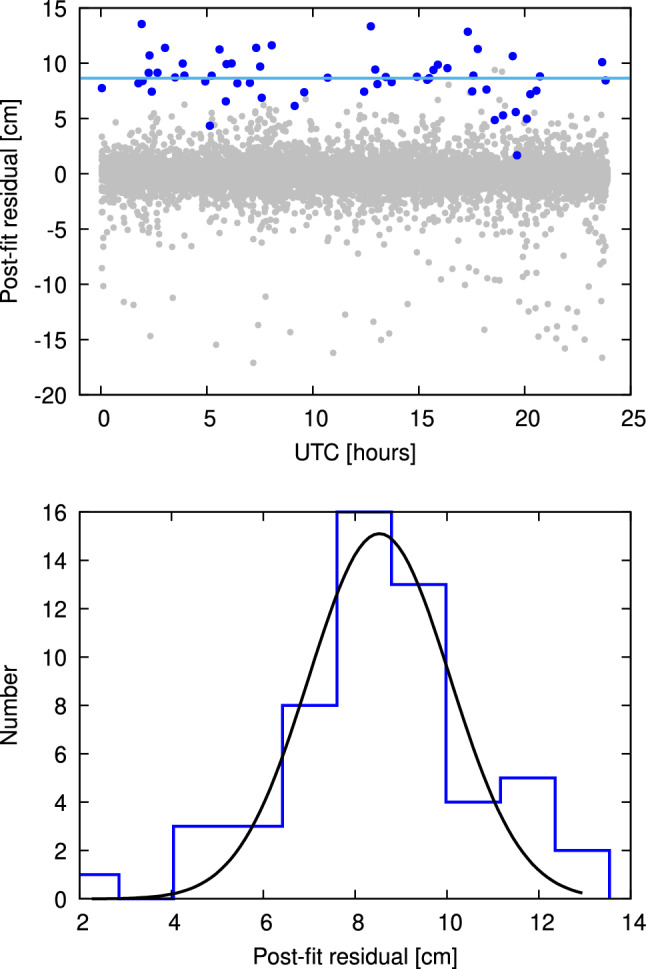
The top panel shows post-fit residuals of the main solution plotted against time. All baselines appear as grey dots except for Kashim11-NyAles20, which is plotted in blue. The solid blue line is the result of fitting the blue data points with a constant, which is determined to be $8.5 \pm 0.3$ cm. The bottom panel depicts a histogram of the post-fit residuals for Kashim11-NyAles20. The distribution is well fitted by a Gaussian centered at $8.5 \pm 1.5$ cm

In the top panel of Fig. 2 we show the post-fit residuals of the VLBI session observed on December 1, 2017. In this plot, which shows the residuals as a function of time, all baselines appear as grey dots except for the baseline Kashim11-NyAles20, which is plotted in blue. The residuals of this baseline are obviously not distributed around zero but show a clear offset in the positive direction. In order to quantify and characterize this offset, we fit the data points with a constant function $f(t) = c$, which results in $c = 8.5 \pm 0.3$ cm, i.e., different from zero with a significance of 28.3$\sigma$. This constant is plotted as the solid blue line in the figure. Introducing a rate does not result in a significant value nor does it improve the fit. We conclude that the bias of the post-fit residuals for this baseline is a constant, significantly different from zero. Furthermore, we investigate the distribution of the residuals of this baseline. The bottom panel of Fig. 2 shows a histogram of the residuals of the baseline. The histogram is well fitted by a Gaussian function $f(r) = A\cdot \mathrm {exp}\left( -0.5(r - r_0)^2/\sigma ^2\right)$ centered at $r_0 = 8.5$ cm with a standard deviation of $\sigma = 1.5$ cm. This example serves to demonstrate that in some cases a baseline-dependent bias in the post-fit residuals which leads to a BCO is obvious immediately. Unfortunately, this is not always the case and conscientious identification and handling of possible BCOs is required to avoid over-parametrization of the solution.

## Estimation of baseline clock offsets

To account for the baseline-dependent clock offset in the VLBI analysis, an extension of the design matrix A is required. The A-matrix consists of partial derivatives of the fundamental geodetic VLBI observable (the group delay $\uptau$) w.r.t. the estimated parameters. The number of columns in the A-matrix equals the number of estimated parameters and the number of rows corresponds to the number of observations, where an observation means a measurement of the group delay at a baseline between two stations.

The total group delay $\uptau _\mathrm{total}$ which is used as input in the geodetic analysis can be represented as the sum of the theoretical modelled delay $\uptau _\mathrm{mod}$ with terms estimated by fitting to the data, such as tropospheric delay $\uptau _\mathrm{trop}$ and clock correction $\uptau _\mathrm{clk}$ arising from the mis-synchronization of the hydrogen maser at each observatory (Cannon 1999). For our purposes, we extend the total group delay by $\uptau _\mathrm{bco}$, which is a delay coming from the baseline-dependent clock offset relative to the reference clock:

3 $$\begin{aligned} \uptau _\mathrm{total} = \uptau _\mathrm{mod} + \uptau _\mathrm{trop} + \uptau _\mathrm{clk} + \uptau _\mathrm{bco}. \end{aligned}$$

The partial derivative of the total group delay with respect to the baseline-dependent clock offset for the particular baseline is thus given by

4 $$\begin{aligned} \frac{\partial \uptau _\mathrm{total}}{\partial \uptau _\mathrm{bco}} = 1. \end{aligned}$$

When the design matrix is augmented for estimating baseline clock offsets, the relationships with the other clock parameters need to be taken care of. In the following, we assume that the interferometer geometry, aberration effects and any noise are already covered in the general estimation process and concentrate only on mean biases of the station clocks and of course the BCOs. The same applies to time variable quantities because the clock behaviour is not a constant and is normally modelled with a drift and higher order terms. However, this is of no importance for the following considerations.

### Selection of reference clock

Estimating station clock parameters can be addressed in the sense of a geodetic datum, similar to a levelling network. In a static situation we have exactly one degree of freedom. This can be considered as the offset of the ensemble of clocks in a network relative to UTC. Ideally, this needs to be determined with picosecond accuracy for all clocks and applied to them. Then the need for estimating clock offsets disappears. In reality, the clock offset of one station is fixed to zero assuming that the correlator setup took care of any known offsets to UTC down to fractions of a microsecond. With the clock datum defined in this way for the reference telescope, any other clock in the network needs to be linked to the reference clock through observations and the respective estimation of relative clock offsets. This step can be considered as the transfer of the clock datum to the *remote* telescopes and is independent of the choice of reference telescope as long as there are observations between the *remote* telescope and one for which the datum had been transferred already.

**Fig. 3:**
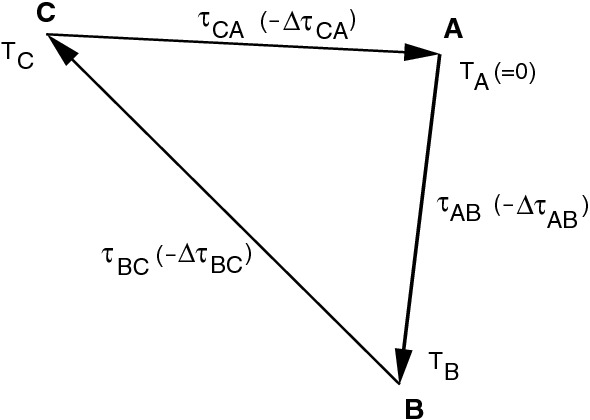
Extension of the schematic Fig. 1 with the wavefront passage epochs at the three stations $T_A$, $T_B$, and $T_C$ and with the baseline clock offsets $\varDelta \uptau _{AB}$, $\varDelta \uptau _{BC}$, and $\varDelta \uptau _{CA}$ at the respective baselines

In this context, it is interesting to see what happens if a different reference clock is chosen. When the reference clock of telescope *A* (Fig. 3) is held fixed, this is equivalent to defining that the wavefront passage epoch $T_A = 0$ which is identical to defining the clock offset being zero. The wavefront passage epoch of telescopes *B* and *C*, $T_B$ and $T_C$, (again equivalent to their clock offsets) can to first order be computed from the entirety of the respective observed delays:

5 $$\begin{aligned} T_B = \uptau _{AB} \end{aligned}$$

and

6 $$\begin{aligned} T_C = - \uptau _{CA}. \end{aligned}$$

Again under the generalizations made above, for Eq. (6) minus Eq. (5) yielding the corresponding total delay of baseline $B \, C$, the delay $\uptau _{BC}'$ closing the triangle has to be computed from the observed total delay $\uptau _{BC}$ taking into account a BCO $\varDelta \uptau _{BC}$:

7 $$\begin{aligned} T_C - T_B = \uptau _{BC}' = \uptau _{BC} - \varDelta \uptau _{BC} . \end{aligned}$$

This is the reason why in fact three parameters have to be estimated, which are $T_B$, $T_C$ and the BCO $\varDelta \uptau _{BC}$, when a BCO is present at the baseline *B* to *C*.

The question now arises whether estimating a BCO $\varDelta \uptau _{CA}$ would yield a different value than that of $\varDelta \uptau _{BC}$ if the reference telescope is changed from *A* to *B* and if there is indeed a BCO $\varDelta \uptau _{BC}$. The short answer is no. The value of a BCO remains the same if we cycle through a triangle with the reference station and the baseline with a BCO opposite of this.

We present two possible ways of explanation. The first argument uses the following logic where the wavefront passage epoch of *B* now is $T_B = 0$. Then the wavefront passage epoch of *A*, $T_A$ results from

8 $$\begin{aligned} T_A = - \uptau _{AB} \end{aligned}$$

and

9 $$\begin{aligned} T_C = \uptau _{BC} = \uptau _{BC}' + \varDelta \uptau _{BC} \end{aligned}$$

because the resulting wavefront passage epoch $T_C$ of *C* is now computed containing the BCO $\varDelta \uptau _{BC}$ and, thus, it has a different value as it was estimated with the observable $\uptau _{CA}$. For the closing baseline *C* to *A* now holds

10 $$\begin{aligned} T_C - T_A = \uptau _{BC}' + \varDelta \uptau _{BC} + \uptau _{AB}. \end{aligned}$$

Estimating $T_C$ and $T_A$ together with $\varDelta \uptau _{CA}$ in fact means that we have estimated a $T_C'$ (fictitious wavefront passage epoch corrupted by the BCO) together with $T_A$ and $\varDelta \uptau _{BC}$.

The second argument follows from Eq. (7) where the wavefront passage epochs $T_B$ and $T_C$ can be described with Eqs. (5) and (6). In this case the BCO $\varDelta \uptau _{BC}$ can be written as:

11 $$\begin{aligned} \varDelta \uptau _{BC} = \uptau _{AB} + \uptau _{BC} + \uptau _{CA} . \end{aligned}$$

Now we rotate the station labels $A \rightarrow B \rightarrow C \rightarrow A$ and Eq. (11) changes to:

12 $$\begin{aligned} \varDelta \uptau _{CA} = \uptau _{BC} + \uptau _{CA} + \uptau _{AB} \end{aligned}$$

where *B* is now the reference station and the only nonzero BCO is on baseline *CA*. It is evident that Eqs. (11) and (12) are equivalent and they also represent the closure delay around this triangle (further details in Sect. 5).

The conclusion is that the value of the estimated BCO stays identical for all baselines in a triangle, independent of its origin and for what baseline it was set up. Please note that from the way the baselines are actually oriented in the triangle, the sign may change. Furthermore, we conclude that estimating BCOs without considering which baselines actually cause BCOs to be nonzero, leads to a correct fit but not to true BCOs which can help to identify technical causes. For a session with many stations, it may be possible to identify stations or baselines primarily responsible for the BCOs by examining which baselines have the largest BCOs. However, this will not work if a bad actor is chosen as the reference station.

For clarity, we include a practical exercise computed with our VLBI software package VieVS (Böhm et al. 2018). In the session 17NOV29XB we exclude all stations except three (*A*: Badary, *B*: Kashim11, *C*: Wettzell) from the analysis to obtain a three station network. Then we run three solutions where each time we change the reference telescope. We apply a very simple parametrization (denoted as SIMPLEPAR) where we estimate one wavefront passage epoch, one rate and one quadratic term at two stations with respect to the reference telescopes, one baseline clock offset, zenith wet delay as piece-wise linear offset every 60 minutes at all three stations and all five EOP as offsets to the a prioris. Table 1 summarizes the estimated wavefront passage epochs ($T_A,T_B, T_C$) and the BCO ($\varDelta \uptau$) at the baseline opposite to the reference telescope from these three solutions confirming the above explanations.

**Table 1:** Estimated wavefront passage epochs w.r.t. a reference telescope (RT) and the BCO in a network of three stations in session 17NOV29XB. Station *A*: Badary, *B*: Kashim11, *C*: Wettzell. The units are picoseconds

Cl. Offset/Reference	Sol.1: RT *A*	Sol.2: RT *B*	Sol.3: RT *C*
$T_A$	0	$-394.6 \pm 43.7$	$97.4 \pm 37.0$
$T_B$	$394.6 \pm 43.7$	0	$575.4 \pm 63.0$
$T_C$	$-97.4 \pm 37.0$	$-575.4 \pm 63.0$	0
	$\varDelta \uptau _{BC} =-83.4 \pm 11.0$	$\varDelta \uptau _{CA} = -83.4 \pm 11.0$	$\varDelta \uptau _{AB} = -83.4 \pm 11.0$

### Selection of baselines for BCO estimation

Another issue to be considered is for which baselines BCOs can be estimated. This is again similar to levelling networks, where the observations might suggest that there are additional biases between any pair of telescopes. For this, a similar approach as for changing the reference station can be applied. Let’s just take again the triangle in Fig. 3. Initially, we estimated $T_B$, $T_C$ and $\varDelta \uptau _{BC}$ through Eqs. (5) and (6) selecting *A* as the reference station with $T_A = 0$. In a slightly different logic, we can determine

13 $$\begin{aligned} T_B = \uptau _{AB} \end{aligned}$$

and

14 $$\begin{aligned} T_C = T_B + \uptau _{BC}. \end{aligned}$$

and estimate the BCO $\varDelta \uptau _{CA}$ from $T_C$ and the observed total delay $\uptau _{CA}$

15 $$\begin{aligned} \varDelta \uptau _{CA} = - (T_C + \uptau _{CA}). \end{aligned}$$

In consequence, in a triangle, we can always estimate one BCO in addition to two *remote* clocks with an arbitrary choice but not more than one.

Another explanation of this fact is that in a general case, from the ensemble of baseline delays for a single triangle, the mean delay for each baseline can be calculated, from which no more than three parameters can be estimated. Those three could be, e.g., three delay differences, or as appropriate to this paper, two wavefront passage epochs and a BCO. Since a BCO is the sum of the three delays [Eqs. (11) and (12)], it is possible to determine only one BCO from the delays in a single triangle.

Returning to the setup of the design matrix, the estimation of BCOs requires additional columns in the A-matrix for each estimated BCO which must not be confused with the columns for estimation of the relative clock offsets per station. Here, the A-matrix is only populated with the value 1 for observations which are carried out on the baseline where the BCO is estimated for. In general it holds, that the maximum number of independent BCO parameters that can be solved for is equal to the number of triangles involving the reference station, i.e. $(n_\mathrm{st}-2)\cdot (n_\mathrm{st}-1)/2$, where $n_\mathrm{st}$ is the number of participating stations in the experiment. But special attention has to be paid to cases where a baseline is dropped or had not been correlated. This can lead to the loss of connection for some *remote* station and estimation of the BCO needs to be reconsidered. This problem can be solved by an exchange of the reference station or by fixing some additional BCOs at more baselines. To be able to solve for the BCOs on a routine basis, an automatic algorithm has to be developed for VLBI analysis software packages, which determines at which baselines the BCOs will be fixed. In VieVS we developed the following procedure for determining a BCO reference telescope (BRT):

- **Step 1** The first choice for the BRT is the telescope which was selected as reference for the clock function (see Eq. (1)). A check follows, whether this telescope has more than $n_{opb}$ observations per baseline to all remaining stations.
- **Step 2** If this is not the case, then a new BRT is selected. This telescope has the highest total number of observations $N_\mathrm{obs}$ and at least $n_{opb}$ observations at all baselines to the remaining stations.
- **Step 3** If there is no such telescope found, the telescope with the highest total number of observations is selected, regardless of the number of observations at individual baselines.

**Fig. 4:**
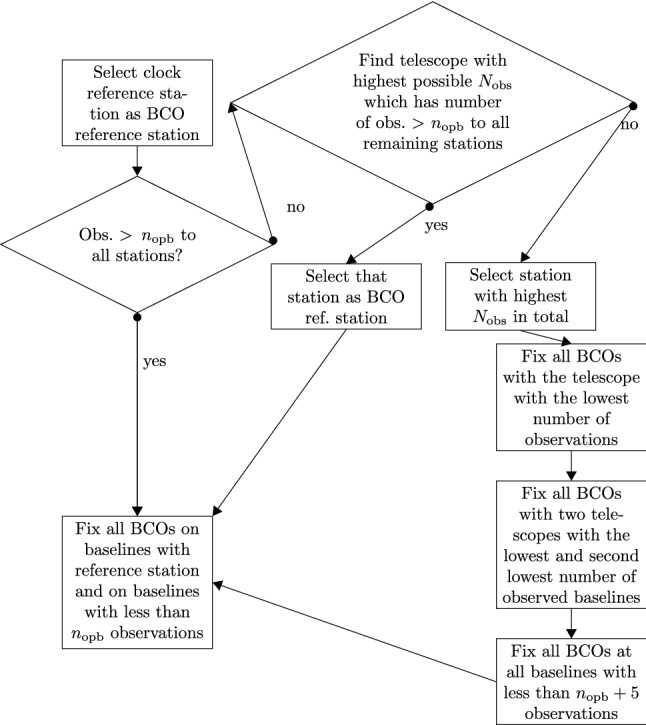
Flowchart of the algorithm to determine the baselines for which BCOs remain fixed, as described in Sect. 3.2

Having identified the BRT (i.e., the reference telescope for the BCO estimation), all baselines including this telescope and baselines with fewer than $n_{opb}$ observations are fixed. At the same time if the Step 3 was necessary, BCOs at the following additional baselines are fixed: (1) at all baselines with the telescope with the lowest total number of observations; (2) at all baselines with the two telescopes with the lowest number of observed baselines (one telescope can be identical with (1)); (3) all baselines with less than $(n_{opb}+10)$ observations in the session.

This algorithm for the baseline selection is visualized in the form of a flowchart in Fig. 4. It was developed during our contribution to the ITRF2020 combination based purely on an empirical approach using $n_{opb} = 5$ proving that the adjustment was not singular for any session. For only 1% of the analyzed sessions (78 out of 6500) we found telescopes with less than five observations to other antennas making Step 3 in this automatic procedure necessary. With this algorithm we identified the large BCOs (larger than three times its formal error) in the dataset and in our final ITRF2020 contribution we estimated only these significant BCOs in the respective sessions.

## Baseline clock offsets in CONT17 sessions

For the assessment of the BCO effect we use data from the continuous VLBI campaign CONT17 observed from November 28 until December 12, 2017 (Behrend et al. 2020). In general, the CONT campaigns are demonstrations of the state of the art of VLBI at a given time. CONT17 differs from the previous CONT campaigns in that there were three independent networks observing. In this paper, data from the two legacy networks are used, denoted as CONT17-L1 and CONT17-L2, scheduled for 14 stations each and observing in S/X frequency bands. For more details about establishing the two global VLBI networks and allocation of the individual stations to the respective legacy networks we refer to Behrend et al. (2020).

Three solutions are computed where the only difference is the list of baselines for which the baseline-dependent clock offset is estimated. In the first solution (SOL1: no BCO), there are no baselines in the list. Therefore, the issue of the BCOs is completely neglected there. In the second solution (SOL2: max BCO), the automatic algorithm (Fig. 4) selecting the baselines is used. In the third solution (SOL3: 3$\sigma$ BCO), only baselines are put on the list where the estimated BCOs in SOL2 are three times larger than their formal error. The telescope serving as reference for the BCO estimation was Badary in CONT17-L1 and BR-VLBA in CONT17-L2.

**Fig. 5:**
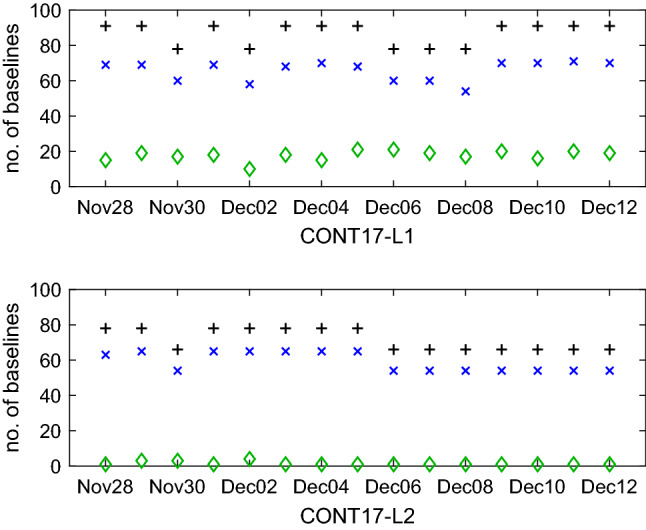
Number of estimated baseline clock offsets in CONT17-L1 (upper plot) and CONT17-L2 (lower plot) networks. Black plus signs show the maximum theoretical number of baselines in the experiment. Blue crosses and green diamonds depict the number of estimated BCOs in solution 2 and solution 3, respectively

**Fig. 6:**
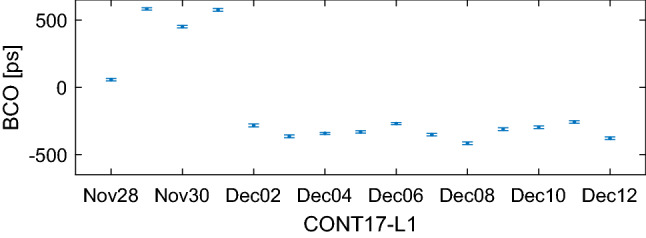
Session-wise estimates of the BCO at baseline Hobart26 - Kashim11 during CONT17 in solution 3

Figure 5 shows the total number of estimated BCOs per session in CONT17 in SOL2 (blue crosses or X’s) and SOL3 (green diamonds). The black plus signs denote the maximum theoretical number of baselines in the given experiment computed as $(n_\mathrm{st}\cdot (n_\mathrm{st}-1))/2$, where $n_\mathrm{st}$ is the number of participating stations in the experiment. Even though both networks were scheduled for 14 stations, the number of participating stations in CONT17-L1 and CONT17-L2 varies between 14-13 and 13-12 stations in each session, respectively, because some stations did not participate in the observation due to technical issues (e.g., SC-VLBA did not participate due to a hurricane damage) or encountered problems during observation period. From the figure it is evident that in the CONT17-L1 network 10-21 baselines in each session do have a significant (larger than three times its formal error (3$\sigma$)) baseline-dependent clock offset. In the adjustment, BCOs up to 18 cm (600 ps) are estimated, therefore CONT17-L1 is a good example of a network where high BCOs occur at several baselines. Figure 6 serves as an example of significant BCOs at one baseline (Hobart26-Kashim11) estimated session-wise in SOL3 during CONT17-L1, showing that the BCO estimates vary from session to session. The detailed causes of this BCO behaviour are under investigation and beyond the scope of this paper. On the other hand, in the CONT17-L2 network no critical (in terms of corrupting the final parameter estimates) baseline-dependent clock offsets are present. In some sessions, 1-4 BCOs larger than 3$\sigma$ are estimated in the adjustment but none of them exceeds 1 cm (33 ps). Therefore, the CONT17-L2 represents for us “ideal” networks where the BCO estimation is not needed. It will help us to answer the question, whether the estimation of BCOs in networks with low or no offsets harms the geodetic solution.

The parametrization of the least-squares adjustment is identical for all three solutions and is as follows: the clock function as sum of quadratic polynomials and PWLO (see Eqs. (1) and (2)) is modelled at all stations w.r.t. the reference clock at station Badary in CONT17-L1 and at station BR-VLBA in CONT17-L2 with 1.3 cm relative constraints between the offsets at 60 min intervals. The station-dependent zenith wet delays are estimated as PWLO every 30 min with 1.5 cm relative constraints and gradients in north and east direction as PWLO every 3 h with 0.5 mm relative constraints. The session-wise station coordinates are estimated for all stations with a no-net-translation and no-net-rotation condition w.r.t. ITRF2014 (Altamimi et al. 2016) and the radio source positions are fixed to ICRF3 (Charlot et al. 2020). The Earth orientation parameters presented in this paper are modelled as PWLO with 1 mas relative constraints and 24 h intervals and adjusted in a global solution of all 15 sessions of the CONT17 networks with stacking and estimation at 0 UT (Krásná 2013).

We apply two different metrics for the assessment of the BCOs. The first metric is the weighted root mean square (wrms) of baseline lengths over the CONT17 campaign together with the wrms of station positions. If an analysis strategy reduces the baseline length scatter, then the VLBI results are more consistent from day-to-day and improved. We also compare the Earth orientation parameters obtained from the three solutions in terms of the wrms. In particular, the pole coordinates are compared against those from the International GNSS Service (IGS). If the agreement is better, then we presume that the VLBI analysis strategy is improved.

**Fig. 7:**
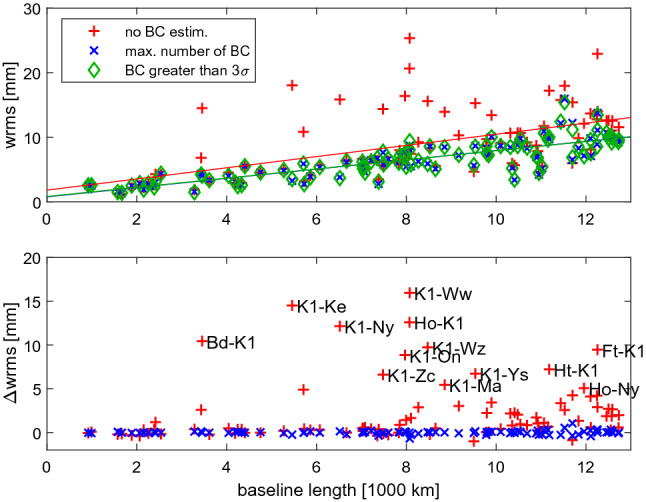
Baseline length scatter (wrms) for CONT17-L1 for the three solutions (upper plot). The lines show the weighted linear fit to the baseline length scatter (green and blue lines overlie each other). Differences in baseline length scatter for CONT17-L1 with respect to the third solution SOL3 are shown in lower plot

**Fig. 8:**
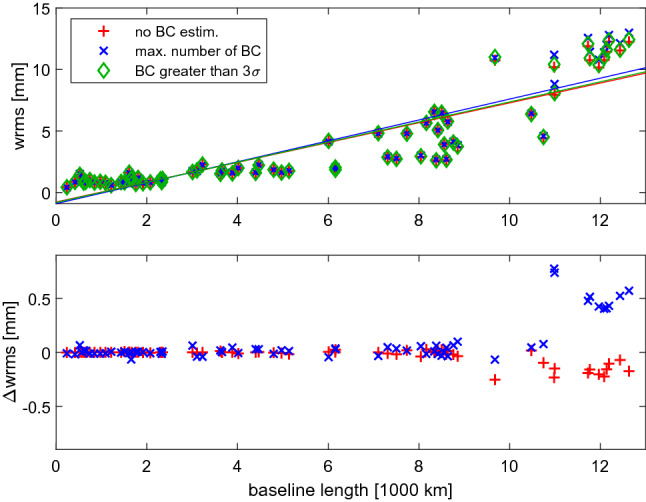
Baseline length scatter (wrms) for CONT17-L2 for the three solutions (upper plot). The lines show the weighted linear fit to the baseline length scatter (all three colors lie close to each other). Differences in baseline length scatter for CONT17-L2 with respect to the third solution SOL3 are shown in lower plot

**Table 2:** Mean improvement in terms of wrms and the percentage of improved (Impr.) baselines and local position components in SOL3 compared to SOL1

	Baseline length		Height component		East component		North component	
	Mean (mm)	Impr. (%)	Mean (mm)	Impr. (%)	Mean (mm)	Impr. (%)	Mean (mm)	Impr. (%)
CONT17-L1	2.2	78.9%	2.3	92.9%	0.6	71.4%	2.1	78.6%
CONT17-L2	− 0.0	28.8%	− 0.0	64.3%	0.0	35.7%	0.0	57.1%

Baseline length repeatability and station position wrms Figures 7 and 8 show the baseline length scatter computed as the wrms from a detrended time series w.r.t. the mean value over the 15 days of CONT17-L1 and CONT17-L2, respectively. In the lower plots, the difference of SOL1 and SOL2 w.r.t. SOL3 is depicted. As noted earlier, CONT17-L1 is a network with large baseline-dependent clock offsets present at several baselines. If the estimation of BCOs in CONT17-L1 is neglected (SOL1) we find high increase of baseline length scatter by up to 16 mm (see Fig. 7). We identified that all twenty baselines with wrms differences above 3 mm in SOL1 w.r.t. SOL3 contain station Kashim11 (K1) or Hobart26 (Ho). For reasons of readability, we added station names only to baselines with wrms differences higher than 5 mm between solutions SOL1 and SOL3. Table 2 summarizes in the first two columns that the length wrms is lower for SOL3 than SOL1 for 78.9% of the baselines in CONT17-L1. The mean improvement of the wrms in SOL3 compared to SOL1 computed over all baselines reaches 2.2 mm.

**Fig. 9:**
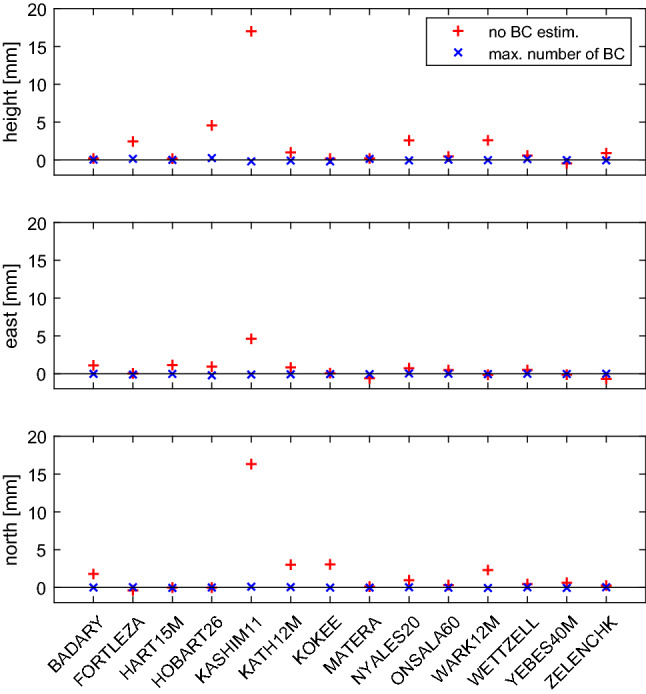
Difference in wrms of the local position components (height, east, north) for stations in CONT17-L1 w.r.t. SOL3

**Fig. 10:**
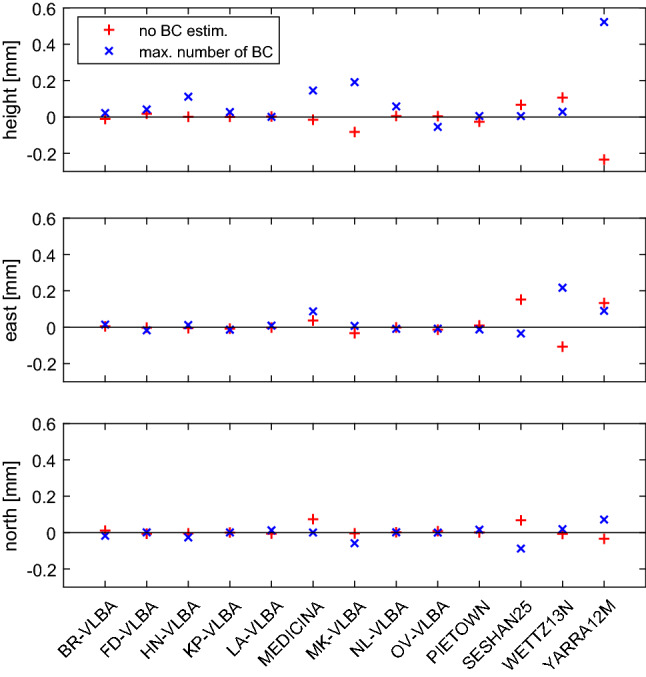
Difference in wrms of the local position components (height, east, north) for stations in CONT17-L2 w.r.t. SOL3

The wrms differences computed for the local (height, east, north) position components for each station in SOL1 w.r.t. SOL3 are plotted with red plus signs in Fig. 9 for CONT17-L1 and summarized in the remaining columns of Table 2.

From the SOL1 and SOL3 for CONT17-L2 (Figs. 8, 10) it is evident that there is not such an improvement if we solve for the BCOs in SOL3 compared to SOL1. Actually, there is a slight increase in the baseline length scatter if BCOs $> 3\sigma$ are estimated in CONT17-L2 as shown in the lower plot of Fig. 8 with the red plus signs for the majority of baselines longer than 9000 km. As noted earlier, in CONT17-L2 there are no critical BCOs in the network, therefore an improvement with SOL3 is not expected. The degradation of the baseline scatter exceeding a limit of 0.1 mm appears at the longest baselines with station Yarra12M only.

**Fig. 11:**
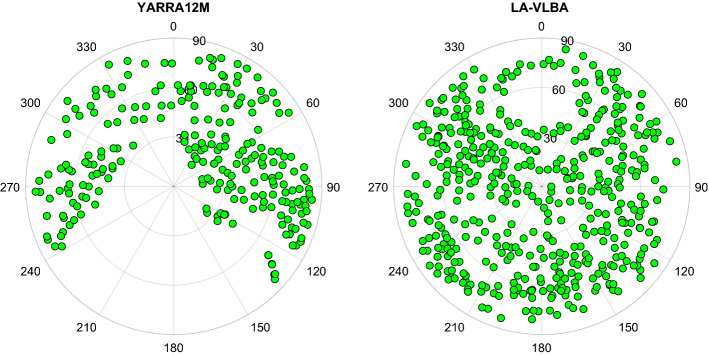
Sky coverage at stations Yarra12M and LA-VLBA during CONT17-L2 in experiment 17DEC12XA

Figure 11 depicts the skyplots with observations for the stations Yarra12M and LA-VLBA for the session on December 12, 2017. According to the CONT17-L2 network design where station Yarra12M is far away from the remaining telescopes, its sky coverage with observations is not optimal. In north-west direction (between azimuths 240$^{\circ }$ and 360$^{\circ }$), the elevation angle upper limit is 60$^{\circ }$, and directions between azimuths 150$^{\circ }$ and 240$^{\circ }$ are without any observation at all. We assume that this causes problems with decorrelation of the station-dependent parameters in the adjustment, such as zenith wet delay, clock offset and station height. If we solve for additional parameters (i.e. BCOs) at the station, the separation of the remaining parameters is even more problematic. This is reflected in the top panel in Fig. 10 where the wrms degradation for the height component of station Yarra12M exceeds 0.2 mm in SOL3 compared to SOL1. For stations with a good sky coverage, e.g., for LA-VLBA as depicted in the right-hand side plot of Fig. 11, the estimation of the BCOs in the network at their baselines changes the wrms of the station position estimates very little, as shown in Fig. 10.

Another comparison, which is depicted in Figs. 9 and 10 with blue X’s, shows the difference in estimated station position wrms if the BCOs are estimated at the maximum number of baselines in the sessions (SOL2) instead only at baselines where the BCOs exceed 3$\sigma$ (SOL3). We want to answer the question whether it is needed to search for the BCOs in the first run and estimate only the significant ones in the second run of the data adjustment. As seen in Table 3, the SOL3-SOL2 differences in mean baseline and mean position component scatter do not exceed 0.1 mm. But even though the mean difference is not very large, the improvement at single stations reaches up to 0.5 mm. This is the case for the height component of Yarra12M in CONT17-L2. In this network a smaller wrms of the height component is seen at 93% of the stations in SOL3 compared to SOL2. Again, the likely cause is that a poor sky coverage does not allow for a proper decorrelation of the station-dependent parameters which implies that for such stations only significant BCOs at their baselines should be estimated to obtain lower wrms for the station height component.

**Fig. 12:**
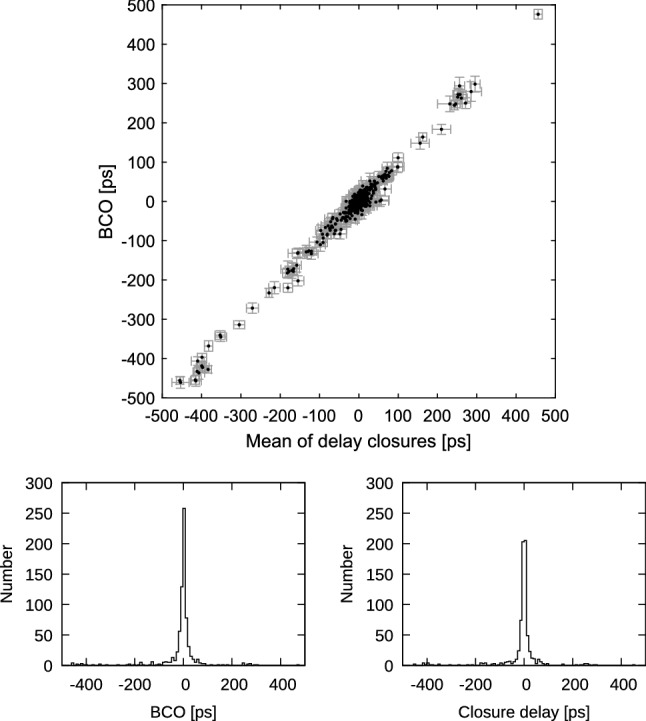
Top: Session-wise baseline clock offsets versus mean of triangle delay closures from ionosphere corrected delay from all CONT17-L1 sessions. Error bars are 1$\sigma$ representations for BCO and rms/$\sqrt{n_{dc}}$ for delay closures, where $n_{dc}$ is number of delay closures in respective triangle. Only triangles with $n_{dc}>$10 are plotted. Bottom left: Histogram of BCOs. Bottom right: Histogram of mean of delay closures

**Table 3:** Mean improvement in terms of wrms and the percentage of improved (Impr.) baselines and local position components in SOL3 compared to SOL2

	Baseline length		Height component		East component		North component	
	Mean (mm)	Impr. (%)	Mean (mm)	Impr. (%)	Mean (mm)	Impr. (%)	Mean (mm)	Impr. (%)
CONT17-L1	0.0	47.8%	0.0	50.0%	− 0.1	14.3%	0.0	57.1%
CONT17-L2	0.1	66.7%	0.1	92.9%	0.0	57.1%	− 0.0	71.4%

**Table 4:** The wrms of EOP from CONT17-L1 and CONT17-L2 w.r.t. 14C04 (w.r.t. IGS finals)

		x-pole [$\mu$as]	y-pole [$\mu$as]	dUT1 [$\mu$s]	dX [$\mu$as]	dY [$\mu$as]
CONT17-L1	SOL1	145 (117)	111 (64)	15.7	64	71
CONT17-L1	SOL2	105 (68)	105 (73)	16.0	52	62
CONT17-L1	SOL3	113 (75)	101 (70)	16.3	62	60
CONT17-L2	SOL1	108 (73)	106 (92)	17.0	68	82
CONT17-L2	SOL2	102 (63)	107 (89)	16.9	62	77
CONT17-L2	SOL3	102 (67)	103 (86)	17.4	66	79

Earth orientation parameters The Earth orientation parameters are computed in a common adjustment of all 15 sessions for each network separately. The estimation interval of the PWLO is set to 24 h with the reference epoch at 0 UT. The station positions are estimated with the no-net-translation and no-net-rotation condition w.r.t. ITRF2014 and the radio source positions are fixed to ICRF3.

Daily estimates of all five EOP in SOL2 and in SOL3 are similar to each other within the formal errors in both networks. Omitting the baseline-dependent clock offsets (SOL1) yields different estimates of the pole coordinates up to 0.2 mas in CONT17-L1 where the BCOs of few cm are present at several baselines as shown in this paper. The corresponding increase of the wrms (in SOL2 and SOL3 relative to SOL1 in CONT17-L1) is about 50% in the x pole component and 10% in the y pole component together with a change in weighted mean value of 60 $\mu$as in both pole components. Table 4 summarizes the wrms of the EOP from all three solutions for both CONT17 networks w.r.t. the a priori IERS 14C04 (Bizouard et al. 2019) time series and the pole coordinates are additionally compared to the IGS finals.^2^

## Baseline clock offsets and triangle delay closures

The analysis of baseline triangle delay closures (i.e. of the sum of delays around a closed triangle of baselines) is another means of investigating the quality of VLBI observations (Herring 1983). With this additional quantity, we infer the hypothesis that a BCO in a triangle is identical to the mean of the triangle delay mis-closures. Taking the mean of the mis-closures of any triangle should average out any source structure effects which inhibit exact closures of individual scans.

**Fig. 13:**
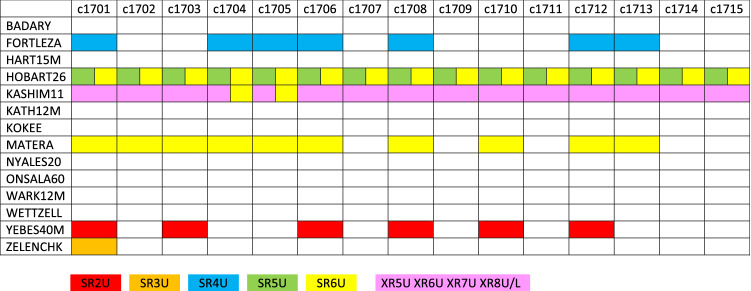
Flagged channels (color-coded) for fringe-fitting according to level-1 processing reports (i.e., block $+$DROP_CHANNELS) in CONT17-L1

The easiest way of verifying this hypothesis is to graphically relate the triangle delay mis-closures to the respective BCOs. For this purpose, we analyse the CONT17-L1 campaign as a three station network to avoid possible propagation of the estimated BCOs to other baselines. We apply the same afore-described simple parametrization SIMPLEPAR of the solution and we set Badary as reference telescope. Each session was analysed $(n_\mathrm{st}-2)\cdot (n_\mathrm{st}-1)/2$ times, i.e., sessions with 14 resp. 13 stations were analyzed 78 resp. 66 times, to build all combinations of triangles with the reference station. From each such solution we obtain the BCO at baseline opposite to the reference telescope. Thereafter, all respective triangle delay mis-closures are formed and the mean value for triangles with more than 10 closure delays during a 24 h session of the CONT17 campaign was calculated. As we can see in the plot of BCOs versus mean of triangle delay closures (Fig. 12), the correlation is striking with correlation coefficient 0.99 and impressively confirms our hypothesis. To accentuate the distribution of the BCOs and mean closure delays we augment the figure with histograms of these values. The two histograms show that the distribution of BCOs (right) is very similar to the distribution of the closure delays (left).

**Fig. 14:**
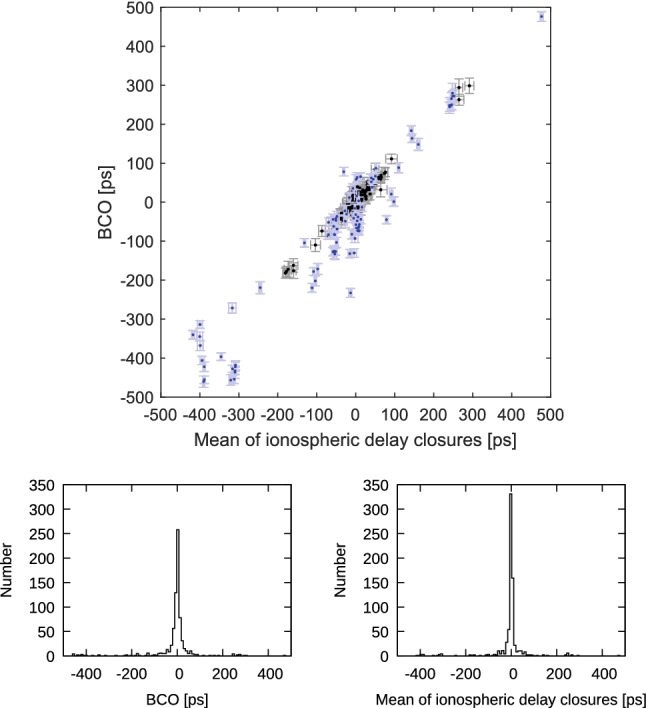
Top: Session-wise baseline clock offsets versus the mean of triangle ionosphere calibration closures from all CONT17-L1 sessions. Triangles with Kashim11 are depicted in blue color. Error bars are 1$\sigma$ representations for BCO and rms/$\sqrt{n_{dc}}$ for delay closures. Only triangles with $n_{dc}>$10 are plotted. Bottom left: Histogram of BCOs. Bottom right: Histogram of mean ionosphere calibration closures

Unfortunately, the graph does not immediately hint at any dominant cause for the triangle delay mis-closures. Performing a few tests, we suspected that discrepancies in the ionospheric corrections may cause the mis-closures, because the level-1 processing reports (fringe fitting) indicated that for some of the telescopes single S band channels had to be discarded due to radio frequency interference or other technical issues (Fig. 13). We then performed the triangle closure computations for the ionospheric delay corrections and compare them to the same BCOs computed from the ionosphere corrected delay as before (Fig. 14). Since Kashim11 has four flagged X band channels for fringe fitting in each session (triangles with Kashim11 are plotted in blue color), the graph (i.e. the comparison between BCO and triangle delay mis-closure from ionospheric delay correction) is noisier than that of Fig. 12. Nevertheless, the correlation coefficient of 0.95 is convincing, proving that it is indeed the ionospheric correction which contributes the dominant part to the delay mis-closures in CONT17-L1.

**Fig. 15:**
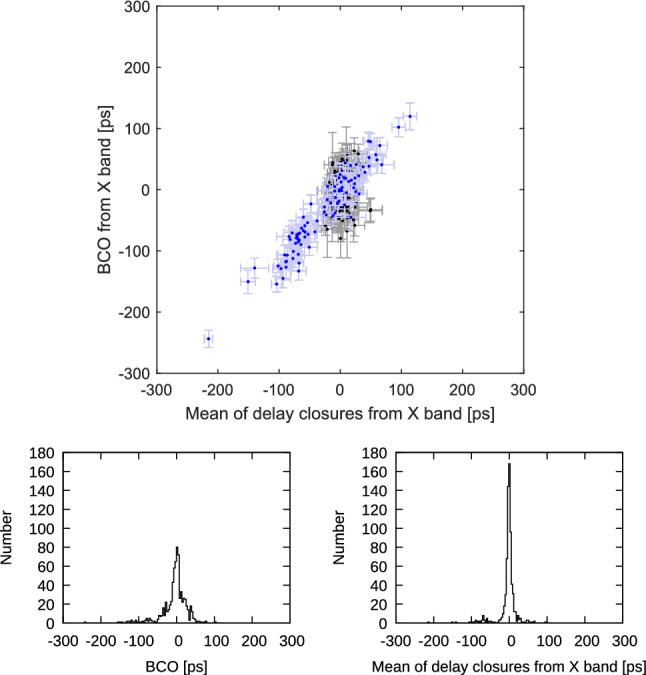
Top: Session-wise baseline clock offsets versus mean of triangle delay closures from X band data of all CONT17-L1 sessions. Triangles with Kashim11 are depicted in blue color. Error bars are 1$\sigma$ representations for BCO and rms/$\sqrt{n_{dc}}$ for delay closures. Only triangles with $n_{dc}>$10 are plotted. Bottom left: Histogram of BCOs from X band. Bottom right: Histogram of mean closure delays from X band

Having identified the most prominent cause for the delay mis-closures and the need for estimating BCOs, we go one step further and look for the floor of the mis-closures vs. BCOs dependency. This can easily be done by running the closure computations and the BCO estimation with the X band data alone. We can do this because the lack of ionosphere calibrations tends to only produce baseline lengths longer than the calibrated ones but leaving any other parameters more or less unaffected. Consequently, this leads to an expansion of the whole network in an isotropic way. Estimating the station coordinates in an unconstrained way with NNR/NNT conditions compensates for the scale extension and, thus, leaves the BCOs unaffected. From the comparison we excluded the Kashim11 data points and depicted them in blue color for a better distinction (Fig. 15). While the scatter in a rms sense of the delay triangle mis-closures narrows to about 8.0 ps, the estimated BCOs reduce to about 20.3 ps. At present we do not have an explanation for the different shapes of the histograms resulting from the X band data alone.

**Fig. 16:**
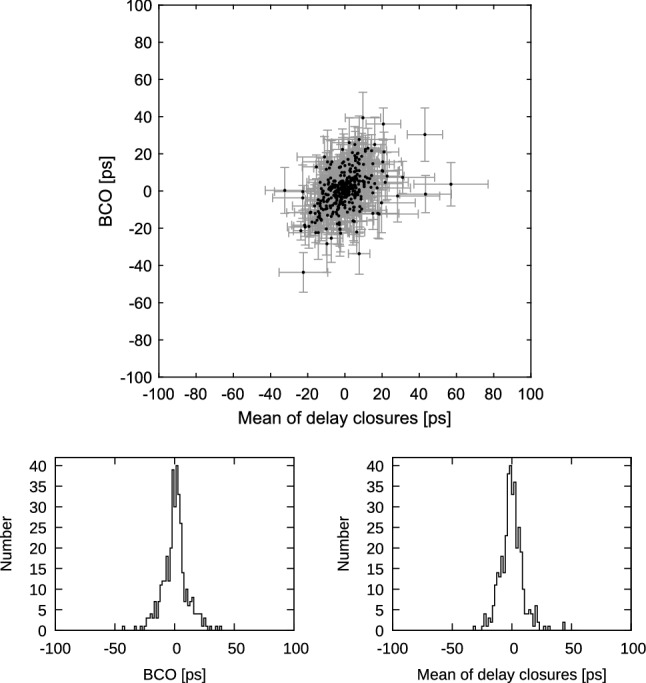
Top: Session-wise baseline clock offsets versus mean of triangle delay closures from final uncorrupted set of observations (triangles with all S and X band channels) in all CONT17-L1 sessions. Error bars are 1$\sigma$ representations for BCO and rms/$\sqrt{n_{dc}}$ for delay closures. Only triangles with $n_{dc}>$10 are plotted. Bottom left: Histogram of BCOs. Bottom right: Histogram of mean of delay closures

**Fig. 17:**
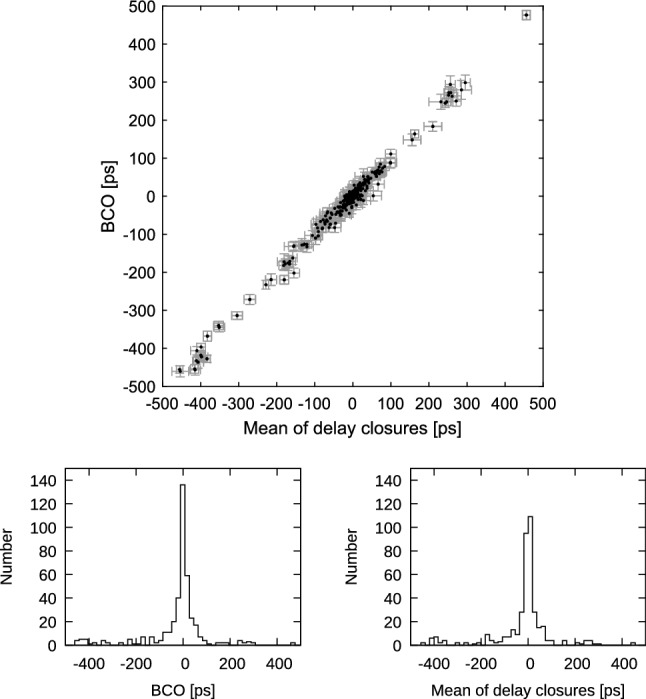
Top: Session-wise baseline clock offsets versus mean of triangle delay closures from the corrupted set of observations (triangles with missing S or/and X band channels) in all CONT17-L1 sessions. Error bars are 1$\sigma$ representations for BCO and rms/$\sqrt{n_{dc}}$ for delay closures. Only triangles with $n_{dc}>$10 are plotted. Bottom left: Histogram of BCOs. Bottom right: Histogram of mean of delay closures

Finally, running a solution with ionospheric calibration and discarding all the data, corrupted by missing channels in S band as well as in X band, the scatter plot shows a remarkable condensation (Fig. 16) with correlation coefficient 0.45. The scatter is 9.8 ps in a rms sense for the ionosphere calibrated triangle mis-closures and 10.4 ps for the respective BCOs. Considering the error level going with the individual data points, we conclude that the relationship between triangle delay mis-closures and BCOs is not random. Figure 17 shows triangles corrupted by missing channels either in S band or X band with correlation coefficient 1.00. Superposing Figs. 16 and 17 yields back Fig. 12 so the condensed knot of Fig. 16 is present in Fig. 12.

## Conclusions

In this publication, we have investigated the effects of baseline clock offsets (BCOs) in VLBI data analysis in a systematic way. We conclude that it is essential to estimate BCOs for baselines where a systematic offset in the observed delay appears. According to our tests, estimation of the BCOs at baselines without any significant offset does not harm the geodetic solution under the condition that there are enough observations at the telescopes with good sky coverage which allow for decorrelation of station-dependent parameters. Therefore, in a routine analysis, we recommend to estimate only the significant BCOs, which are larger than three times their formal error. We showed that the nominal value of the estimated BCO in a triangle stays constant, no matter for what baseline it was set up. We conclude that estimating BCOs with an arbitrary reference telescope leads to a correct fit in the adjustment but not to true BCOs in the observing network. Further we confirm that a BCO in a triangle is identical to the mean of the triangle delay mis-closures within the analysis uncertainty. It was recognised that the dominant effect for the occurrence of significant BCOs comes from the ionospheric delay calibration. As soon as the delay determination originates from a fringe fitting process of uncorrupted data of all eight X band and all six S band channels, the spread in rms sense in BCOs and triangle delay mis-closures is 10 ps only.

## Data Availability

All observation data were retrieved from publicly available database. The software package VieVS which is necessary to reproduce the results is open access and can be downloaded from https://github.com/TUW-VieVS/.

## Footnotes

1 https://sourceforge.net/projects/nusolve/.

2 International GNSS Service, GNSS Final Combined Earth Rotation Parameter (ERP) Product, Greenbelt, MD, USA:NASA Crustal Dynamics Data Information System (CDDIS), Accessed January 11, 2021 at 10.5067/GNSS/gnss_igserp_001.
